# 维生素C对A549细胞增殖、凋亡及Caspase-3、Survivin表达的影响

**DOI:** 10.3779/j.issn.1009-3419.2010.02.01

**Published:** 2010-02-20

**Authors:** 鹏勇 翟, 锦荣 曾, 宁 谭, 绩英 王, 岚珍 黄, 巍巍 佘

**Affiliations:** 541000 桂林, 桂林医学院附属医院呼吸内科 Department of Respiritory, Affilited Hospital of Guilin Medical University, Guilin 541000, China

**Keywords:** 维生素C, 肺肿瘤, 细胞凋亡, Caspase-3, Survivin, Vitamin C, Lung carcinoma, Apoptosis, Caspase-3, Survivin

## Abstract

**背景与目的:**

维生素C作为一种抗氧化剂, 对多种肿瘤均有抑制作用, 本研究旨在探讨维生素C对肺癌细胞株A549的增殖、凋亡的影响及其诱导A549细胞凋亡的可能机制。

**方法:**

在体外培养的肺癌A549细胞株中加入不同浓度的维生素C, 采用细胞生长曲线及克隆形成实验检测细胞生长情况; 用流式细胞仪检测细胞周期的影响及凋亡率; 用RT-PCR方法检测肺癌细胞株A549中Caspase-3、Survivin的表达差异。

**结果:**

400 μg/mL、4 mg/mL浓度组维生素C明显抑制A549细胞的增殖, 流式细胞仪检测细胞被阻止在G_0_/G_1_期及S期, 且随着时间的延长细胞凋亡逐渐增多, RT-PCR检测维生素C可以上调Caspase-3 mRNA的表达, 并且随着时间的延长Caspase-3 mRNA的表达逐渐增强, 对Survivin mRNA的表达无确切作用。

**结论:**

维生素C呈时间和剂量依赖性抑制A549细胞的增殖, 并使A549细胞阻止在G_0_/G_1_期及S期, 并呈时间依赖性诱导A549细胞凋亡, 其机制可能是通过上调Caspase-3的表达。

## Abstract (en)

**Background and objective:**

It was proven that Vitamin C could inhibit the growth of many types of tumors as an antioxidant.The aim of this study is to explore role of Vitamin C in proliferation and apoptosis of lung carcinoma cell line A549 and the underlying mechanism.

**Methods:**

A549 cells were cultured *in vitro* and incubated with Vitamin C.The cell viability was measured by growth curve and clonogentic assay.Flow cytometry was used to analyze cell cycle and detect apoptosis.The levels of expression of Caspase-3 mRNA and Survivin mRNA were detected by RT-PCR.

**Results:**

Vitamin C of 400 μg/mL, 4 mg/mL significantly inhibited the growth of A549 cell lines (*P*=0.024, *P*=0.015, respectively).Flow cytometry showed that the cells major stagnation stayed in the G_0_/G_1_ and S phase and the apoptotic rate increased with time prolonged.Vitamin C signifiantly up-ragulated the expression of Caspase-3 mRNA, but had no effect on Survivin mRNA.

**Conclusion:**

Vitamin C can inhibit the proliferation of A549, block A549 cells in G_0_/G_1_ and S phase, and induce apoptosis of A549 cells.Apotosis occurred by up-ragulated the expressionof Caspase-3.

维生素C(Vitamin C, Vit C)是一种具有6个碳原子的酸性多羟基化合物。其分子中2位和3位碳原子的两个烯醇式羟基极易解离, 释放出H^+^, 而被氧化成脱氢Vit C。Vit C和脱氢Vit C在人体内形成可逆的氧化还原系统, 此系统在生物氧化、还原作用及细胞呼吸中起重要作用。Vit C作为细胞保护剂广泛应用于临床多种疾病(如坏血病、肝硬化等)的治疗。近年来, 研究^[1]^发现Vit C可以促进肿瘤细胞的凋亡, 但具体机制尚未完全阐明。本研究将探讨Vit C对肺癌A549细胞株的增殖、凋亡的影响及其可能机制。

## 1 材料和方法

### 1.1 实验材料与实验分组

#### 1.1.1 实验材料

肺癌细胞株A549由中山大学附属肿瘤医院惠赠, DMEM培养液(GIBICOL公司), 小牛血清(GIBICOL公司), Vit C(广州南国药业有限公司), CCK-8试剂(大连宝生物有限公司), RT-PCR反应体系(Promega公司), Caspase-3 mRNA及Survivin mRNA引物由上海赛百盛生物技术有限责任公司合成。

#### 1.1.2 实验分组

以50×D/5 000×2×10^3^公式^[2]^计算出Vit C临床用药量为400 μg/mL。实验分为对照组及40 μg/mL、400 μg/mL、4 mg/mL三种浓度Vit C组。

### 1.2 实验方法

#### 1.2.1 细胞培养

A549细胞置于含10%小牛血清、100 U/mL青霉素、100 μg/mL链霉素的DMEM培养液中, 在37 ℃、5%CO_2_培养箱中传代培养。每两天换一次液, 4 d传代。

#### 1.2.2 细胞增殖测定(CCK-8法)

取指数期A549细胞, 待生长至近融合状态, 经0.25%EDTA的PBS消化后, 配成5 000/mL, 然后将细胞以1 000/孔接种于96孔板, 置于CO_2_培养箱培养24 h后加药, 在培养箱中继续培养24 h、48 h、72 h、96 h、120 h、144 h、168 h后, 加入10 μL CCK-8混合液, 震荡10 s, 继续培养2 h后吸出上清液450 nm测OD值, 绘制生长曲线。

#### 1.2.3 细胞集落形成测定(克隆形成法)

取对数生长期的A549细胞, 用0.25%胰蛋白酶消化并吹打成单个细胞, 离心后, 把细胞重悬在10%胎牛血清的DMEM培养液中备用。将细胞悬液稀释成500个/mL, 以1 000/孔接种于6孔板中, 并轻轻转动, 使细胞分散均匀。置37 ℃、5%CO_2_培养箱培养1周, 当培养皿中出现镜下可见的克隆时, 加入以上浓度药物培养1周。终止培养。弃去上清液, 用PBS小心浸洗2次。加纯甲醇5 mL, 固定15 min。然后去固定液, 加适量结晶紫应用染色液染30 min, 然后用流水缓慢洗去染色液, 空气干燥。将平皿倒置并叠加一张带网格的透明胶片, 用肉眼直接计数克隆, 并在显微镜(低倍镜)计数大于20个细胞的克隆数。

#### 1.2.4 流式细胞术检测细胞周期及细胞凋亡率

用含10%胎牛血清的DMEM培养液调整细胞浓度为1×10^6^/mL接种于10 cm皿中, 置于5%CO_2_、37 ℃培养箱中培养12 h后, 用含0.5%小牛血清的DMEM培养液继续培养24 h, 使细胞周期同步化。加入Vit C(400 μg/mL), 对照组不加药, 分别作用6 h、12 h、24 h、48 h, 收集贴壁细胞, 制成单细胞悬液, 70%乙醇固定, 4 ℃保存过夜, -20 ℃保存(有效期为1周)。检测前用PBS洗去固定液, 加入20 μL RnaseA, 37 ℃孵育30 min后, 暗处加PI染液, 冰浴30 min, 染色后以300目筛网过滤。调整细胞浓度为1×10^5^/mL-1×10^6^/mL, 采用流式细胞仪检测, 激发光源为氩离子, 激发波长488 nm, 用Multicycle DNA分析软件行细胞周期的测定。

#### 1.2.5 RT-PCR检测Caspase-3 mRNA、Survivin mRNA的表达水平

取对数生长期A549细胞加入400 μg/mL的Vit C作用6 h、12 h、24 h、48 h后TRIzol一步法提取总RNA, 取1 μL总RNA以10 μL体系逆转录, 制备cDNA, 并稀释30倍备用。Caspase-3 mRNA上游引物:5’-TGACCGAGGCTACATTCAGATGACACC-3’, 下游引物:5’-CAAGAGAGTTGGGCTGACCAGAAACAC-3’, 扩增产物365 bp; Survivin mRNA上游引物:5’-CCCTTTCTCAAGGACCACCGCATC-3’, 下游引物:5’-CACTGAGAACGAGCCAGACTTGGC-3’, 扩增产物133 bp; β-actin上游引物:5’-AGTGTGACGTGGACATCCGCA-3’, 下游引物:5’-ATCCACATCTGCTGGAAGGTGGAC-3’, 扩增产物243 bp。扩增后取扩增产物用琼脂糖凝胶电泳检测。应用凝胶成像系统对电泳条带进行扫描分析, 计算相对表达水平(与β-actin比值)。

#### 1.2.6 统计分析

所有实验均重复3次, 用Mean±SD表示, 经SPSS 10.0进行两两比较的*t*检验, *P* < 0.05为有统计学差异。

## 2 结果

### 2.1 细胞增殖情况及生长曲线

以40 μg/mL、400 μg/mL、4 mg/mL三种浓度Vit C干预A549抑制增殖情况见图 1, 结果表明400 μg/mL Vit C、4mg/mL Vit C明显抑制A549的增殖(*P* < 0.05), 而40 μg/mL Vit C抑制作用不明显(*P* > 0.05);随着浓度的增大, 3天抑瘤率明显增大, 见图 2。

**1:**
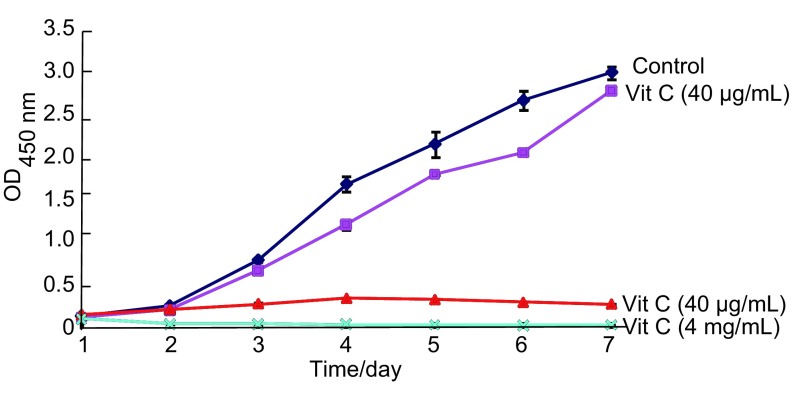
不同浓度Vit C作用A549细胞生长曲线 Cell proliferation curve of A549 cell with Vit C at different doses

**2:**
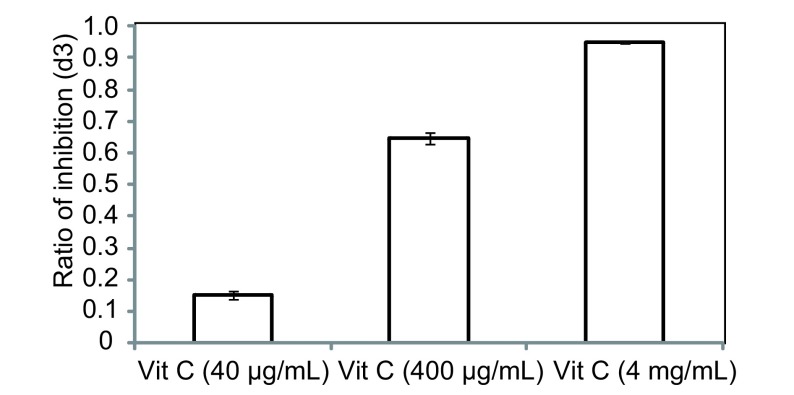
不同浓度Vit C作用A549瘤生长抑制率(第3天) Ratio of inhibition of A549 cell with Vit C at different doses (d3)

### 2.2 细胞集落形成观察

A549细胞分别加入40 μg/mL VitC、400 μg/mL Vit C、4 mg/mL Vit C, 肉眼克隆见图 3A, 镜下计数见图 3B, 400 μg/mL Vit C、4mg/mL Vit C组明显抑制了肺癌A549细胞克隆的形成, 与对照组、40 μg/mL Vit C组相比有统计学差异(*P* < 0.05)。

**3:**
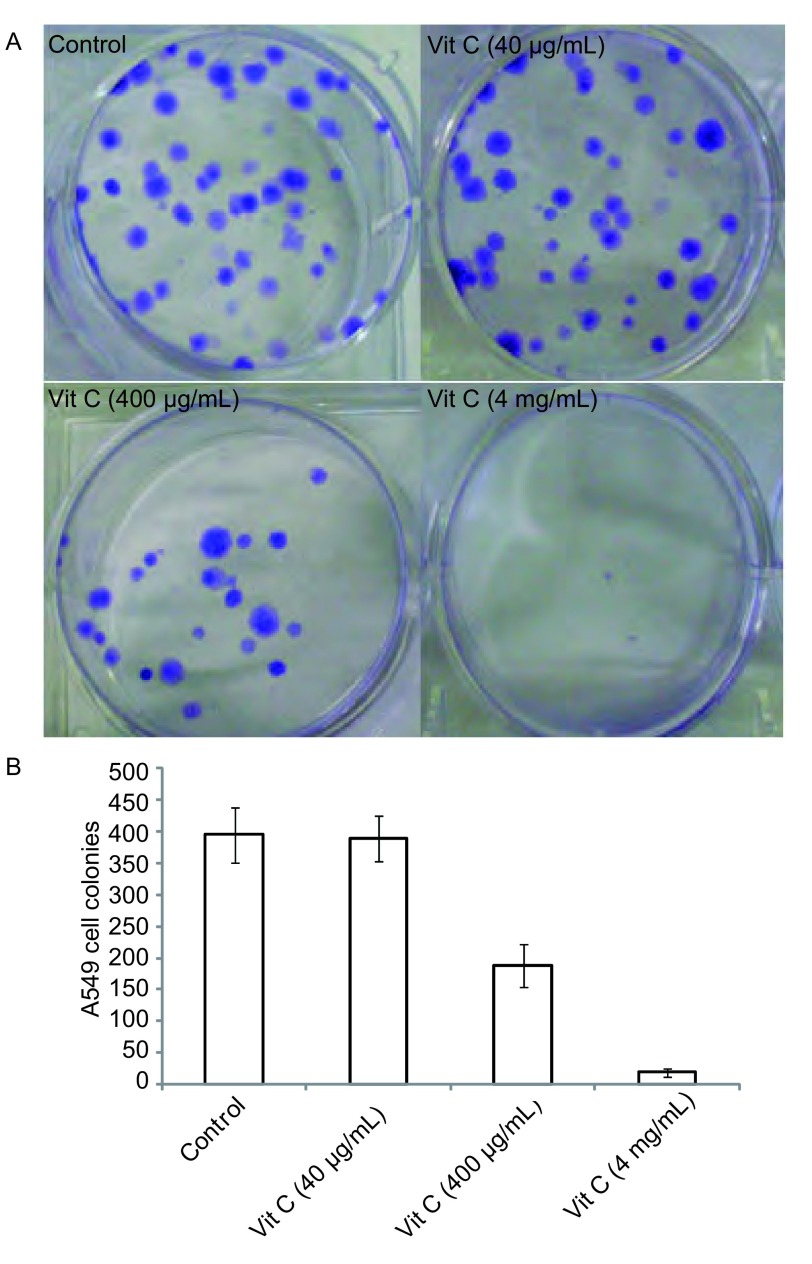
不同浓度Vit C作用A549后集落形成数 Colony of A549 cell with Vit C at different doses

### 2.3 流式细胞仪检测细胞周期及凋亡实相结果

图 4可见, 临床用药量(400 μg/mL)的Vit C随着作用时间的延长, G_0_/G_1_期、S期的细胞明显增多, 进入G_2_/M期的细胞明显减少, 说明Vit C可以延长A549的细胞周期, 从而抑制其增殖, 当作用24 h、48 h后G_0_/G_1_期、S期的细胞明显增多, 并且随着作用时间的延长细胞凋亡数明显增加。

**4:**
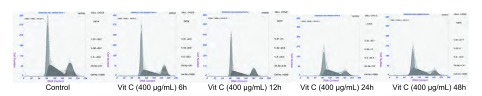
400 μg/mL Vit C干预A549细胞不同时间后流式细胞仪检测凋亡和细胞周期变化结果 Changes of cell cycle and apoptosis after 400 μg/mL Vit C treated A549 cell different time

### 2.4 RT-PCR检测Vit C对Caspase-3 mRNA、Survivin mRNA水平的影响

取对数生长期A549细胞, 加入400 μg/mL的Vit C培养6 h、12 h、24 h、48 h后提取总RNA进行RTPCR, 扩增产物用琼脂糖凝胶电泳检测, 结果表明Vit C作用6 h后Caspase-3 mRNA的表达增高, 随着作用时间的延长表达量无明显增多(*P*=0.001、0.002、0.002、0.003);随着Vit C作用时间的延长, Survivin mRNA的表达并未明显变化(*P*=0.959、0.552、0.23、0.173), 见图 5。

**5:**
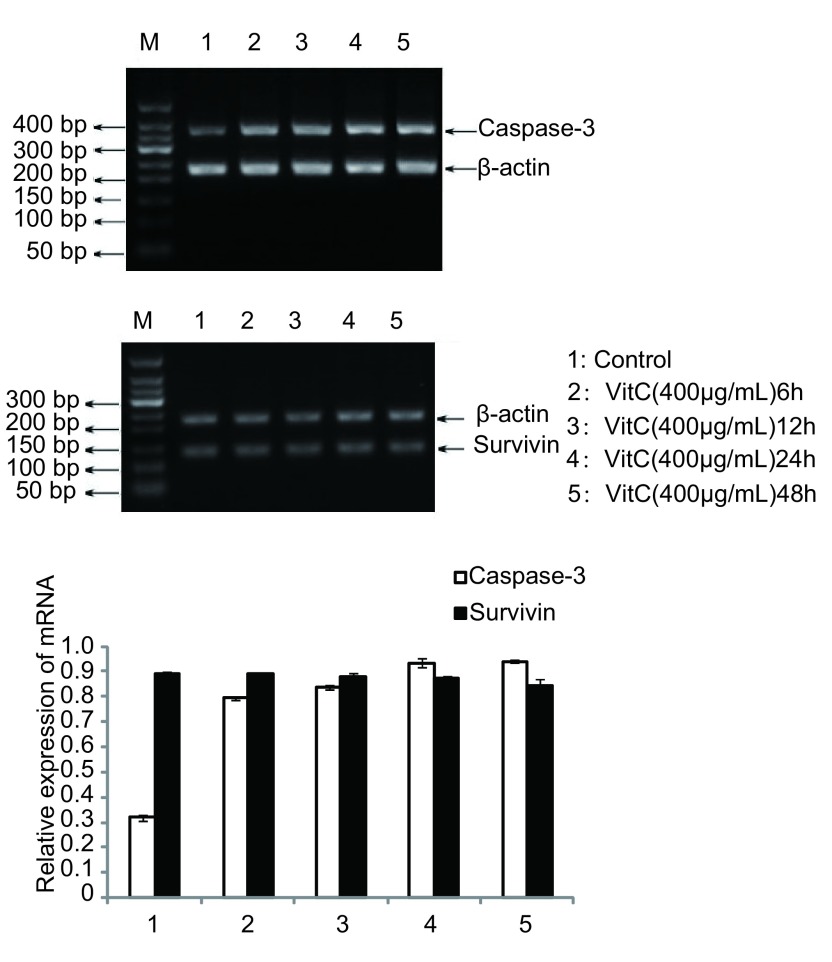
400 μg/mL Vit C干预A549细胞不同时间对Caspase-3 mRNA、Survivin mRNA表达的影响 Expression of Caspase-3 mRNA, Survivin mRNA after 400 μg/mL Vit C treated A549 cell different time

## 3 讨论

Vit C是具有许多生物学功能的水溶性己糖衍生物, 它的分子式是C_6_H_8_O_6_, 其分子中2位和3位碳原子的两个烯醇式羟基极易解离, 释放出H^+^。通常情况下, 我们普遍认为小剂量Vit C是天然抗氧化剂, 通过还原作用清除氧自由基, 而大剂量Vit C具有氧化作用^[3-4]^。而研究也主要集中于Vit C在小剂量时对肿瘤发生的预防作用, 对肿瘤的治疗作用的研究还处于初始阶段。本研究发现Vit C在400 μg/mL时即可以明显抑制肺癌A549细胞的增殖(*P* < 0.05), 流式细胞仪检测发现400 μg/mL Vit C可以将A549细胞阻滞在G_0_/G_1_期及S期, 使进入G_2_/M期的细胞明显减少, 从而延长了细胞周期, 并且发现随着作用时间的延长, 可以明显促进A549细胞的凋亡。

众所周知, 凋亡逃逸是肿瘤发生、发展的重要机制之一。细胞凋亡主要通过线粒体通路、死亡受体通路和内质网通路启动凋亡。线粒体通路主要是含BH3结构域的Bcl-2家族成员与另外的Bcl-2家族成员(Bax亚家族成员Bax、Bak)等作用, 导致后者的寡聚并插入线粒体膜, 引起线粒体膜通透性改变, 跨膜电位丢失, 释放细胞色素C(Cyt C)和其它蛋白^[5]^, Cyt C在ATPPdATP存在的情况下, Cyt C与凋亡蛋白酶活化因子(apoptotic protease activating factor, Apaf-1)形成多聚复合体, 通过Apaf-1氨基端的Caspase募集结构域(caspase recruitment domain, CARD)募集胞质中的Caspase-9前体, 并使其自我剪切活化并启动Caspase级联反应, 激活下游的Caspase-3和Caspase-7, 完成其相应底物的剪切, 引起细胞凋亡^[6]^; 死亡受体通路主要机制是死亡配体与死亡受体激活无活性的pro-caspase-8变为有活性的Caspase-8。激活的Caspase-8不仅可激活下游效应Caspase裂解多种蛋白质而最终导致细胞凋亡, 同时还可以降低线粒体内膜电位使Bc1-2家族成员裂解, 导致Cyt C的释放^[7]^, 后者可与Apaf-1结合, 在dATP的存在下活化Caspase-9, Caspase-9能激活下游效应Caspase-3^[8]^。而Caspase-3作为这两条通路上的下游共同基因, 在细胞凋亡过程中起着至关重要的作用。本研究发现, 随着作用时间的延长Vit C可以上调Caspase-3的表达, 提示Vit C可能通过氧化作用进一步减少肿瘤细胞内本来就缺乏的过氧化物酶, 并增加过氧化氢(H_2_O_2_)的产生^[9]^从而作用于pro-caspase激活Caspase-3诱导A549细胞凋亡。而作为最强的凋亡抑制基因, Survivin在肿瘤逃逸凋亡过程中有着不可忽视的作用, 可直接与Caspase-3、Caspase-7结合并抑制它们的活性^[10]^而阻止肿瘤细胞凋亡的发生。本研究亦观察了Vit C对Survivin的影响, 发现其并不明显下调Survivin mRNA的表达。因此我们认为, Vit C可能虽然未能下调Survivin mRNA的表达, 但可能阻止Survivin蛋白与Caspase-3的结合, 从而影响肿瘤细胞的凋亡过程。综上所述, 我们认为在临床治疗量的Vit C即可以明显抑制肺癌A549的增殖, 并将其阻滞在G_0_/G_1_期及S期, 并且随着作用时间的延长, 可以明显诱导肺癌A549的凋亡, 其机制可能是通过上调Caspase-3的表达。因此, 我们有理由相信Vit C作为肿瘤化疗的辅助用药在临床应用中有广阔的前景。
